# Computational Neuroscience

**DOI:** 10.1155/2014/120280

**Published:** 2014-03-06

**Authors:** Jinde Cao, Qingshan Liu, Sabri Arik, Jianlong Qiu, Haijun Jiang, Ahmed Elaiw

**Affiliations:** ^1^Department of Mathematics, Southeast University, Nanjing 210096, China; ^2^Department of Mathematics, Faculty of Science, King Abdulaziz University, Jeddah 21589, Saudi Arabia; ^3^School of Automation, Southeast University, Nanjing 210096, China; ^4^Department of Computer Engineering, Istanbul University, Avcilar, 34320 Istanbul, Turkey; ^5^College of Science, Linyi University, Linyi, Shandong 276005, China; ^6^College of Mathematics and System Sciences, Xinjiang University, Urumqi 830046, China

This special issue of Computational Neuroscience (CN) includes 15 original articles, which have been selected from the highest quality submissions. The articles present the state-of-the-art research on the theory and applications of computational models and methods. Among the articles, 6 ones are oriented to systems and analysis, whereas the remaining 9 ones deal with algorithms and applications in diverse areas.

The articles for systems and analysis are the combination of theoretical approaches and practical problems, namely, to formulate mathematical system reasonably from true environment. The 6 articles deal with the first aspect from different areas in great depth. Y. Qi et al. proposed a higher-order coupling neural network model including the inhibitory neurons and investigated its dynamic behaviors, titled *“The effect of inhibitory neuron on the evolution model of higher-order coupling neural oscillator population.”* The global stability of a human immunodeficiency virus infection model was studied by A. M. Elaiw et al. in article *“Global stability of HIV infection of CD4+ T cells and macrophages with CTL immune response and distributed delays.”* J. Li et al. investigated the perturbation analysis of a linear system titled *“On optimal backward perturbation analysis for the linear system with skew circulant coefficient matrix.”* L. Ni et al. proposed a method with dynamic extension by introducing a shifted time window in their article titled *“Analyzing EEG of quasi-brain-death based on dynamic sample entropy measures.”* The detailed behaviors of large neuronal networks on parallel computer systems were studied by L. L. Pesce et al. in *“Large-scale modeling of epileptic seizures: scaling properties of two parallel neuronal network simulation algorithms.”* Y. Jin et al. discussed the bifurcation behaviors of systems in their article *“Bifurcations and stability of nondegenerated homoclinic loops for higher dimensional systems.”*

The 9 articles dealing with the second aspect of algorithms and applications are based on different engineering problems. N. Zhou et al. investigated the fuzzy c-means clustering algorithms for medical image segmentation, titled *“An improved FCM medical image segmentation algorithm based on MMTD.”* The article *“A 3D finite-difference BiCG iterative solver with the Fourier-Jacobi preconditioner for the anisotropic EIT/EEG forward problem” *authored by S. Turovets et al. introduced and explored the performance of several new promising numerical techniques for solving the investigated problems. For observing human brain activations in real time, the real-time functional magnetic resonance imaging technique was investigated in *“Incremental activation detection for real-time fMRI series using robust Kalman filter”* by L. Li et al. The article by J. Zhu and M. Ren proposed a novel image mosaic method based on scale invariant feature transform of line segment for image processing. The article titled *“The research and application of visual saliency and adaptive support vector machine in target tracking field”* by Y. Chen et al. investigated the application of ASVM for target tracking. The article by Y. Chen et al. with title *“The study of randomized visual saliency detection algorithm”* investigated the image segmentation process for high quality visual saliency map. The article titled *“A semantic medical multimedia retrieval approach using ontology information hiding”* by K. Guo and S. Zhang investigated an effective semantic medical multimedia retrieval approach which can reflect the users' query intent. For the analysis of brain decoding with functional magnetic resonance imaging, L. Wang et al. investigated the feature analysis in article *“Principal feature analysis: a multivariate feature selection method for fMRI data*.*”* The face recognition was investigated in *“Membership-degree preserving discriminant analysis with applications to face recognition”* by Z. Yang et al. with a novel feature extraction algorithm.

We sincerely hope that our efforts by compiling these articles can enrich our readers and inspire researchers with regard to the seemingly common but actually important issue of CN.

*Jinde Cao* *Jinde Cao* *Qingshan Liu* *Qingshan Liu* *Sabri Arik* *Sabri Arik* *Jianlong Qiu* *Jianlong Qiu* *Haijun Jiang* *Haijun Jiang* *Ahmed Elaiw* *Ahmed Elaiw*

